# Ecologic, Geoclimatic, and Genomic Factors Modulating Plague Epidemics in Primary Natural Focus, Brazil

**DOI:** 10.3201/eid3009.240468

**Published:** 2024-09

**Authors:** Matheus F. Bezerra, Diego L.R.S. Fernandes, Igor V. Rocha, João L.L.P. Pitta, Natan D.A. Freitas, André L.S. Oliveira, Ricardo J.P.S. Guimarães, Elainne C.S. Gomes, Cecília Siliansky de Andreazzi, Marise Sobreira, Antonio M. Rezende, Pedro Cordeiro-Estrela, Alzira M.P. Almeida

**Affiliations:** Instituto Aggeu Magalhães, Fiocruz, Brazil (M.F. Bezerra, D.L.R.S Fernandes, I.V. Rocha, J.L.L.P. Pitta, E.C.S. Gomes, M. Sobreira, A.M. Rezende, A.M.P. Almeida);; Laboratório de Mamíferos, Pós Graduação em Ciências Biológicas (Zoologia), Universidade Federal da Paraíba, João Pessoa, Brazil (N.D.A. Freitas, P. Cordeiro-Estrela);; Núcleo de Geoprocessamento, Instituto Aggeu Magalhães, Fiocruz (A.L.S. Oliveira);; Laboratório de Geoprocessamento, Instituto Evandro Chagas, Belém, Brazil (R.J.P.S. Guimarães);; Laboratório de Biologia e Parasitologia de Mamíferos Silvestres Reservatórios, Instituto Oswaldo Cruz, Fiocruz, Rio de Janeiro, Brazil (C.S. de Andreazzi);; Universidad Complutense de Madrid, Madrid, Spain (C.S. de Andreazzi);; International Platform for Science, Technology and Innovation in Health, PICTIS, Ílhavo, Portugal (C.S. de Andreazzi);; Group of Biotechnology Applied to Pathogens, René Rachou Institute, Fiocruz (A.M. Rezende)

**Keywords:** Plague, Yersinia pestis, bacteria, parasites, zoonoses, vector-borne infections, climate, rainfall, ecological network, Brazil, One Health

## Abstract

Plague is a deadly zoonosis that still poses a threat in many regions of the world. We combined epidemiologic, host, and vector surveillance data collected during 1961–1980 from the Araripe Plateau focus in northeastern Brazil with ecologic, geoclimatic, and *Yersinia pestis* genomic information to elucidate how these factors interplay in plague activity. We identified well-delimited plague hotspots showing elevated plague risk in low-altitude areas near the foothills of the plateau’s concave sectors. Those locations exhibited distinct precipitation and vegetation coverage patterns compared with the surrounding areas. We noted a seasonal effect on plague activity, and human cases linearly correlated with precipitation and rodent and flea *Y. pestis* positivity rates. Genomic characterization of *Y. pestis* strains revealed a foundational strain capable of evolving into distinct genetic variants, each linked to temporally and spatially constrained plague outbreaks. These data could identify risk areas and improve surveillance in other plague foci within the Caatinga biome.

Plague is a deadly bacterial disease caused by *Yersinia pestis* and is implicated in major pandemics throughout the course of human history. Despite the decline in global incidence, plague outbreaks still occur in regions where the bacterium maintains a sylvatic cycle (*1*,*2*). In addition, plague resurgence has been reported after long periods of quiescence, making active animal surveillance in its natural foci critical to prevent outbreaks in human populations (*3*).

Plague incidence typically undergoes accentuated variations over time (*2*). Evidence from studies performed in central Asia and North America demonstrate that such variations are triggered by the combined effect of geoclimatic and ecologic factors. Temperature, precipitation, landscape, vegetation, soil composition, and host–vector densities and diversity have been reported to interplay in a trophic web that leads to plague dissemination in both wild fauna and humans (*3*–*5*). However, information regarding the role the geoclimatic and ecologic aspects in the modulation of plague systems in foci of Brazil is lacking.

The current demarcation of plague focal areas in Brazil lacks granularity and encompasses extensive regions solely based on geologic formations with reported plague cases (*6*). Thus, identification of specific geoclimatic and environmental patterns indicating hotspots for increased plague risk could provide valuable information for implementing more assertive and targeted animal surveillance.

The Araripe Plateau (Chapada do Araripe) constitutes a geologic landmark situated along the border of Pernambuco, Ceará, and Piauí states of Brazil. Although the region is in the core of the semiarid biome known as Caatinga, the plateau is topped by savanna, and its slopes have lush forest vegetation because of orographic precipitation and a permeable tabletop (*7*,*8*). The Araripe Plateau was considered the epicenter of plague in Brazil until 1980, when the last confirmed human case in the region was notified (*9*).

We took advantage of the geographically well-delimited features of the Araripe Plateau and the robust amount of data obtained from the Pilot Plague Program (*10*) regarding human and animal surveillance to perform a case study of a plague focus in Brazil through a One Health perspective. By combining 20 years of epidemiologic data with ecologic, geoclimatic, and *Y. pestis* genomic variables, we aimed to unravel the intricate dynamics of plague in a natural focus in Brazil.

## Methods

### Data Collection

**Figure 1:**
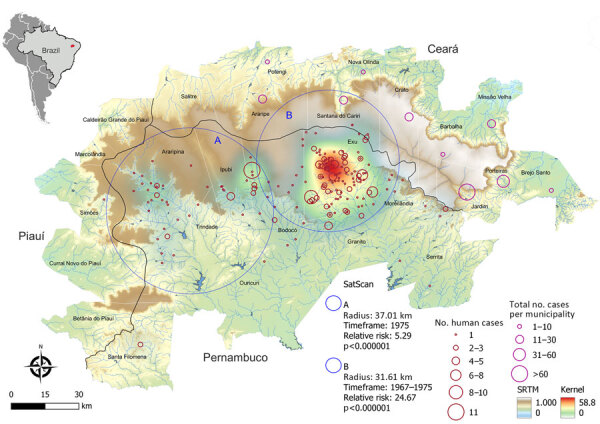
Spatial distribution and risk analysis of human plague cases in a study of ecologic, geoclimatic, and genomic factors modulating plague epidemics in primary natural focus, Brazil. Background colors show the altimetry (m) from SRTM. The black line shows the tri-state boundaries between Pernambuco, Ceará, and Piauí. Red circles identify plague risk areas by application of KDE in human cases in Pernambuco. Blue circles A and B indicate plague risk clusters calculated by SaTScan for 1975 (A) and 1967–1975 (B). Pink circles indicate spatial distribution of human plague cases by number of occurrences per municipality in Ceará. Inset map shows Brazil with the Araripe Plateau focus in red. KDE, kernel density estimation; SaTScan, space-time scan (https://www.satscan.org) statistics; SRMT, Shuttle Radar Topography Mission (https://www.earthdata.nasa.gov).

In brief, we retrieved information on *Y. pestis* reservoirs, vector surveillance, and notification of human plague cases from the national Brazilian Plague Reference Service (https://www.cpqam.fiocruz.br/sr/peste) document repository. We obtained metadata from the Fiocruz *Y. pestis* collection of the World Data Centre for Microorganisms (https://www.wdcm.org; collection no. 1040) and the CONCEPAS database (http://cyp.fiocruz.br). We collected demographic and climatic variables from multiple public databases (Appendix 1 Figure 1).

### Geospatial Analysis

We collected coordinates from human cases where public health conducted site visits. To identify plague risk hotspots, we calculated the kernel density estimation (KDE) and space-time scan (SaTScan, https://www.satscan.org) statistics. We interpolated rainfall data from meteorologic stations by using inverse weighted distance and used the normalized difference vegetation index (NDVI) as a proxy for vegetation coverage (Appendix 1).

### Laboratory Testing for Plague

Human plague diagnosis was performed through a combination of clinical and epidemiologic assessment and laboratory testing, such as culturing bubo aspirates or blood cultures. Rodent diagnoses were made by direct microscopy observation of spleen imprints and blood smears, followed by conventional bacteriology. Fleas also were tested via bacterial culturing (Appendix 1). 

### Statistical Analysis and Ecologic Networks

We calculated linear regression, nonlinear correlations, principal component analysis (PCA) and receiver operating characteristic curves by using GraphPad Prism version 10.1 (GraphPad, https://www.graphpad.com). We analyzed the fundamental properties of each host–vector plague network by using the bipartite package in R (The R Project for Statistical Computing, https://www.r-project.org). We considered p<0.05 statistically significant.

### Whole-Genome Sequencing and Bioinformatic Analysis

We performed DNA extraction by using the DNeasy Blood & Tissue Kit (QIAGEN, https://www.qiagen.com). We conducted genomic library preparation using the Nextera XT Library Preparation Protocol (Illumina, https://www.illumina.com) and used the MiSeq sequencer and 600-cycle version 3 cartridge (Illumina) for sequencing. We assembled genomes by using VelvetOptimiser and annotated by using the Prokka pipeline, as previously described (*11*). We conducted core single-nucleotide variant (SNV) calling by using Snippy (https://github.com/tseemann/snippy) and used IQ-TREE2 (http://www.iqtree.org) to perform phylogenetic analyses based on the core SNV. We then visualized results by using the iTOL (https://itol.embl.de) platform.

## Results

### Spatiotemporal Features of Plague Outbreaks in the Araripe Plateau Region

**Figure 2:**
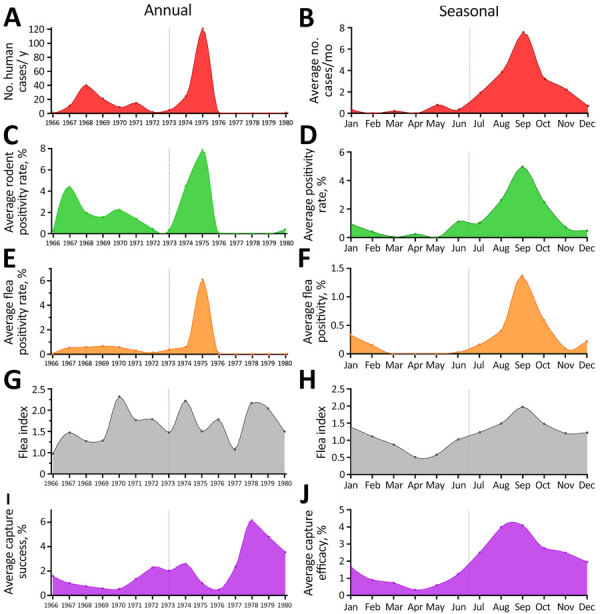
Annual (left column) and seasonal (right column) dynamics of plague occurrence and flea and rodent capture and abundance rates in a study of ecologic, geoclimatic, and genomic factors modulating plague epidemics in primary natural focus, Brazil. A, B) Human cases; C, D) rodent positivity; E, F) flea positivity; G, H) flea index (number of fleas per host); I, J) rodent capture success. Vertical lines provide midpoints for comparison between measured indices.

During 1961–1980 in the Araripe Plateau focus, 551 human plague cases were reported; 292 were in the state of Pernambuco, 240 in the state of Ceará, and only 1 in the state of Piauí (Appendix 1 Figure 2, panels A, B). However, we excluded 18 cases in Pernambuco from the spatial analysis because case locations were unknown.

Human plague cases were concentrated mostly at the foothills of the Araripe Plateau, in regions of lower altitude, but close to the plateau’s slope (Figure 1). KDE analysis concentrated the plague risk more intensely in 3 areas of lower altitude in the municipalities of Exu, Bodocó, and Araripina, and those areas are closed in by concave cliffs (Figure 1). Conversely, only a few cases were reported on the top of the plateau. To overcome bias of heterogeneous population density, we performed a SaTScan analysis using the census tracts as geographic areas, which confirmed the findings from the KDE analysis. Furthermore, the estimated relative risk for the population living within the radius of 2 areas were much higher than for the surrounding areas; 5.29-fold higher in area A and 24.67-fold higher in area B (Figure 1). The year-by-year dynamics of plague cases in the region were clearly visible (Video).

**Video:** Series of human cases a study of ecologic, geoclimatic, and genomic factors modulating plague epidemics in primary natural focus, Brazil.

### Effect of Eco-Epidemiologic and Climatic Variables on the Dynamics of Human Plague Cases

**Figure 3:**
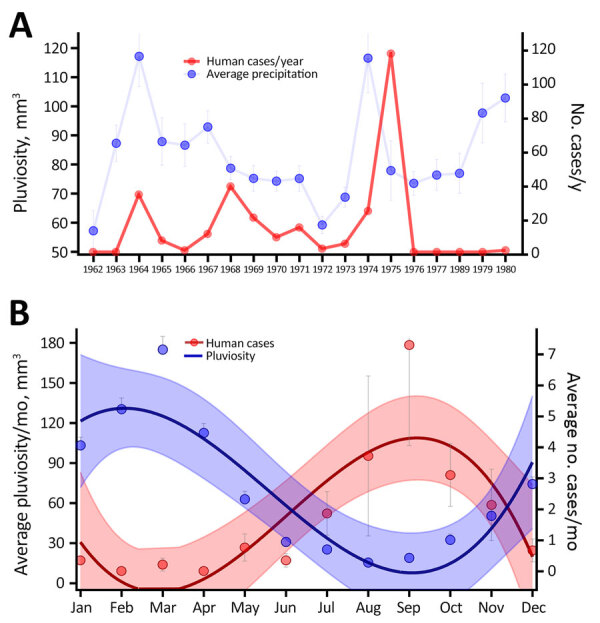
Effects of climatic variables on human cases in a study of ecologic, geoclimatic, and genomic factors modulating plague epidemics in primary natural focus, Brazil. A) Yearly average pluviosity (rainfall amount); B) monthly average pluviosity. Dots indicate averages; whiskers indicate upper and lower limits. Average pluviosity (mm^3^) was measured in municipalities in the state of Pernambuco in the Araripe Plateau region during 1962–1980. The curves in panel B represent the third order polynomial interpolation of cases and pluviosity averages; shaded areas indicate 95% CIs.

By analyzing precipitation levels and data from 40,972 rodents and 39,150 fleas tested for *Y. pestis* in the Araripe Plateau region during 1966–1980, we were able to assess the seasonal and annual effect of those variables on the number of human cases. Overall, the years with higher rodent and flea *Y. pestis* positivity rates overlapped the years with more human cases (Figure 2, panels B, D, F, H, J). In addition, using rodent capture success as a proxy for animal abundance, we found that years with more human cases and *Y. pestis–*positive animals also had reduced rodent abundance, indicating that *Y. pestis* circulation had a major effect on the rodent population. Of note, the annual dynamics of average pluviosity (rainfall amount) showed a 1-year delay effect when compared with human cases until 1976 (Figure 3, panel A), after which plague became quiescent in the region. Thus, we considered the pluviosity from the previous year in further analyses.

Next, we evaluated the seasonal aspects of those variables by measuring the average monthly rates for several additional variables during 1966–1980. Along with the human cases, rodent and flea *Y. pestis* positivity rates, flea index (number of fleas per host), and rodent abundance all showed a strong seasonal component, peaking during the end of winter and beginning of spring­ (August­–October) (Figure 2). We noted that rainfall occurred mostly in the first semester, peaking during late summer and early fall (February–April) (Figure 3, panel B).

**Figure 4:**
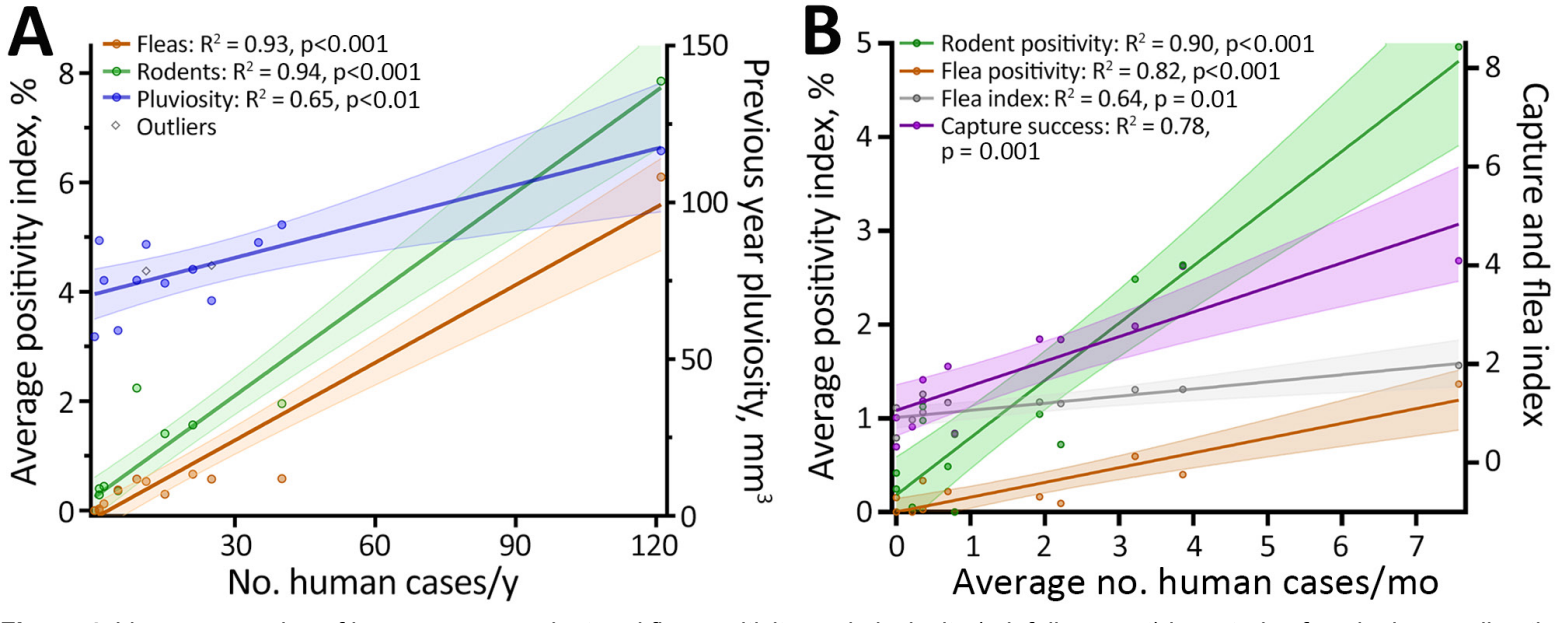
Linear regression of human cases, rodent and flea positivity, and pluviosity (rainfall amount) in a study of ecologic, geoclimatic, and genomic factors modulating plague epidemics in primary natural focus, Brazil. Annual (A) and monthly (B) average number of human cases compared with *Yersinia pestis* positivity among rodents and fleas and average pluviosity are shown. The previous year pluviosity data only included years from the plague outbreaks, 1966–1976. Solid lines indicate averages, shaded areas indicate 95% CIs, and dots indicate outliers.

**Figure 5:**
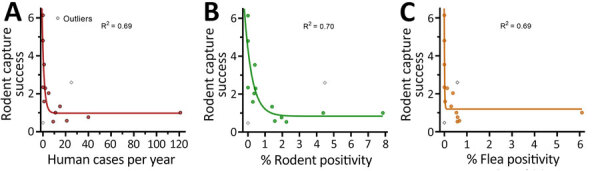
Exponential correlation between rodent capture success and human and animal *Yersinia pestis* positivity in a study of ecologic, geoclimatic, and genomic factors modulating plague epidemics in primary natural focus, Brazil. A) Human cases; B) rodent positivity; C) flea positivity. Capture success serves as a proxy for rodent abundance.

The occurrence of human cases was proportionally related to rodent (R^2^ = 0.94) and flea (R^2^ = 0.93) *Y. pestis* positivity rates, and human cases related more moderately (R^2^ = 0.65) to the previous year’s pluviosity (Figure 4, panel A). We also ran those variables in a multivariable regression model; however, rodent and flea positivity were also mutually dependent and therefore redundant (R^2^ = 0.94) (Appendix 1 Figure 2, panels C, D). For that reason, the model was better explained by a single variable linear regression. Linear regression of flea index showed no impact of this variable on human cases or rodent infection. By comparing monthly averages during 1966–1980, we found that the rodent abundance (capture success), flea index, and rodent and flea *Y. pestis* positivity rates had a stark linear proportionality with human cases at a seasonal level (Figure 4, panel B). Of note, although the effect of rodent and flea *Y. pestis* positivity rates on human cases showed a linear pattern, rodent abundance had a negative exponential correlation with plague activity in humans, and *Y. pestis* positivity in animal hosts and vectors (Figure 5).

**Figure 6:**
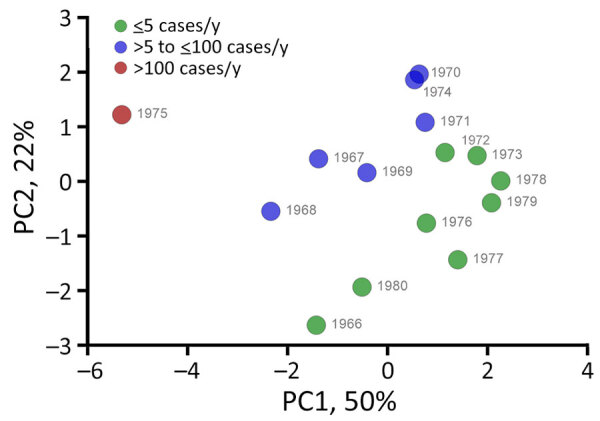
Year-based principal component analysis in a study of ecologic, geoclimatic, and genomic factors modulating plague epidemics in primary natural focus, Brazil. PC based on eco-epidemiologic and climatic features. The weight of each included variable is provided in Appendix 1 Figure 2, panel E. PC, principal component.

The PCA of multiple eco-epidemiologic and climatic features revealed that distinct years clustered together according to the intensity of human cases, suggesting a synergic effect among those variables. Of note, 1975, which had the highest number of human cases, showed unique eco-climatic features. Those features included lower abundance of *Necromys lasiurus* mice; distinct ecologic network parameters, such as host–vector robustness and modularity; high precipitation (considering a 1-year lagged effect); and higher *Y. pestis–*positivity rates in rodents and vectors (Figure 6; Appendix 1 Figure 2, panel E). We designated years as epidemic or low-activity according to the PCA analysis.

### Spatiotemporal Dynamic of Human Plague Cases by Rainfall and Vegetation Coverage

**Figure 7:**
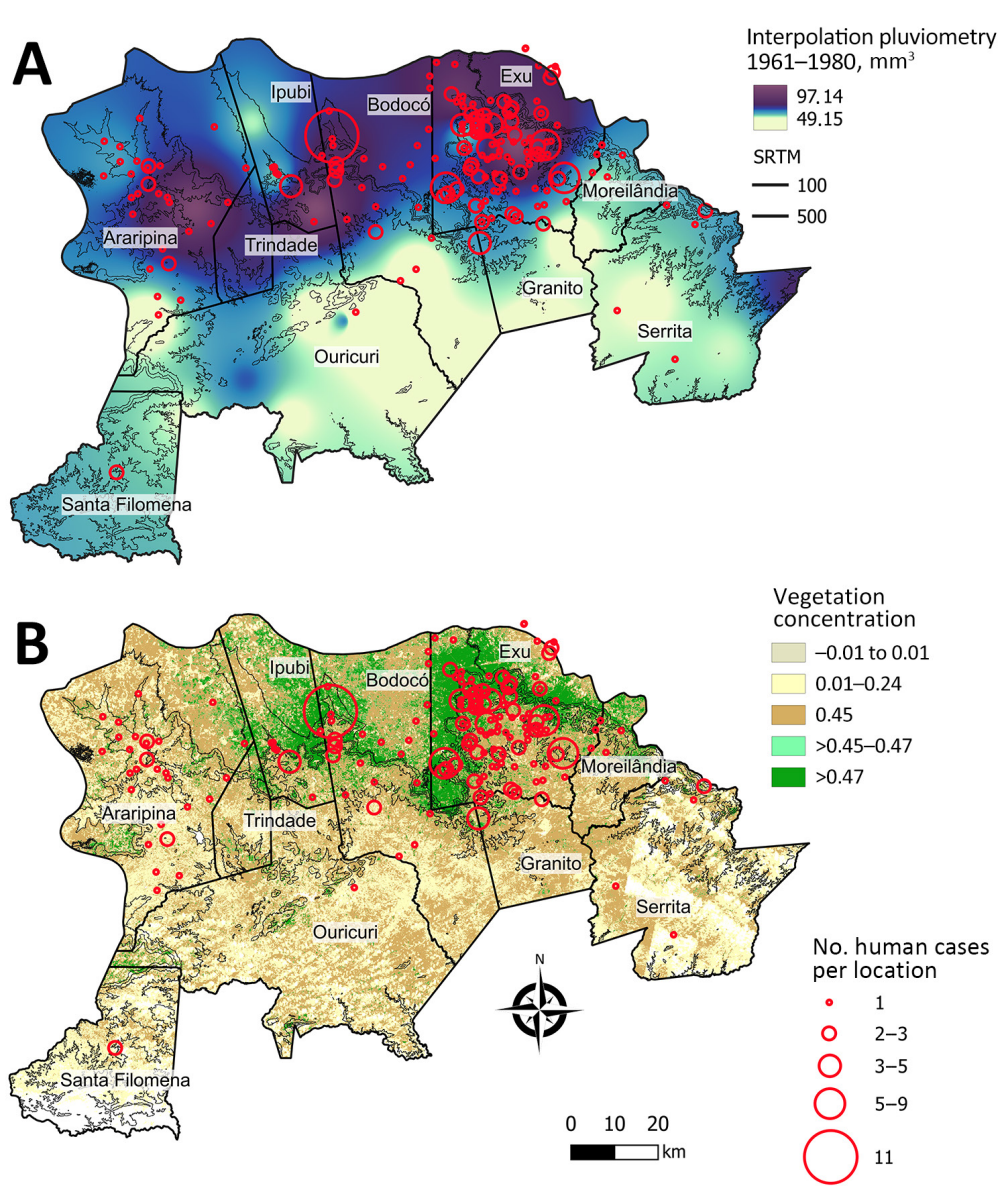
Spatial distribution of rainfall (pluviometry) and vegetation coverage in a study of ecologic, geoclimatic, and genomic factors modulating plague epidemics in primary natural focus, Brazil. A) Locations of human cases and interpolation of average pluviometry (1961–1980) in the Araripe Plateau municipalities of Pernambuco state. B) Locations of human cases and normalized difference vegetation index model showing vegetation concentrated in the slopes of the Araripe Plateau, overlapping the main rainfall and plague case areas. Images obtained by the Landsat 4 satellite in 1984.

On the basis of our preliminary findings that human plague cases correlated with the average pluviosity from the previous years in the Araripe Plateau region (Figure 3, panel A), we expanded our analysis to evaluate whether that effect would also demonstrate a spatial pattern. By interpolating pluviometry data from meteorologic stations in the region, we observed a marked overlap between clusters of plague cases and rainfall volume during the study period (Figure 7, panel A). Similar to the plague cases, rain was more intense in lower altitude areas neighboring the elevated plateau. Next, we also investigated the spatial distribution of rainfall in the years that anticipated major outbreaks (1967 and 1974) or epidemiologic silence (1972) and observed a 1-year delay in spatial overlap between rainfall and plague cases (Appendix 1 Figure 3).

Considering the semiarid climate in the region, we hypothesized that one of the mechanisms through which rainfall could modulate plague would be effects on the availability of vegetation as a food source for wild rodents. NDVI data showed that vegetation was concentrated in the slopes of the Araripe Plateau, overlapping the main rainfall and plague case areas (Figure 7, panel B).

### Ecologic Aspects of Plague in Araripe Plateau and Ecologic Networks

**Figure 8:**
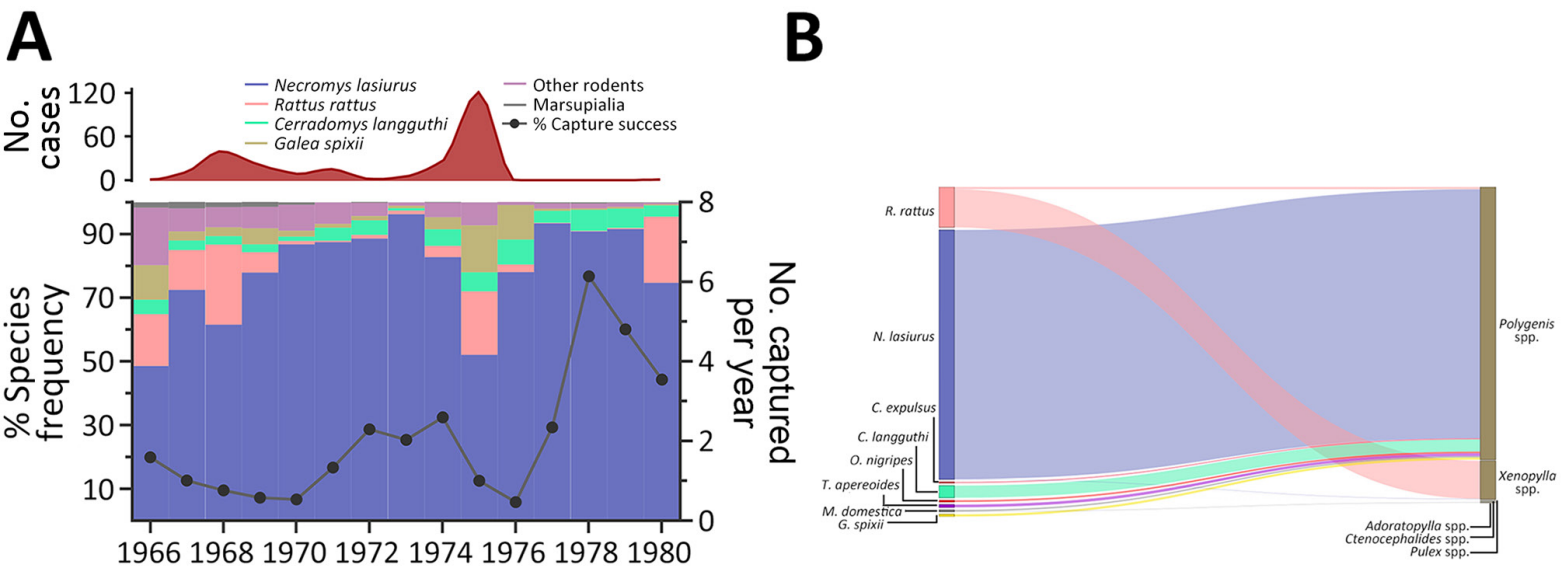
Diversity of rodent and flea species during distinct epidemiologic scenarios of plague in a study of ecologic, geoclimatic, and genomic factors modulating plague epidemics in primary natural focus, Brazil. A) Frequency of each rodent species on capture traps per year (left axis), yearly capture success (right axis) and human plague cases (red curve on top). B) Sankey diagram representing the distribution of rodents and other small mammal hosts according to the flea species and frequency.

*N. lasiurus* mice were the most representative rodent species during the study period. Nevertheless, *N. lasiurus* mice representation varied through time and was greatly reduced in epidemic years (Figure 8, panel A). Moreover, reduction of *N. lasiurus* mice populations had a dramatic effect on the overall capture success rate. Of note, after the abrupt shift from a major plague outbreak in 1975 to quiescence, the wild rodent population quickly reestablished. 

Among flea vector species, the most common in captured rodents was *Polygenis* spp. fleas, which were the predominant and almost exclusive species in wild rodents, including *N. lasiurus* mice. On the other hand, *Xenopsylla* spp. fleas were mostly found in the synanthropic *Rattus rattus* rats. Less commonly, *Adorapsylla* spp. and *Ctenocephalides* spp. fleas were also identified (Figure 8, panel B). Because the method of flea species classification changed over time, we referred to fleas only at the genus level.

**Figure 9:**
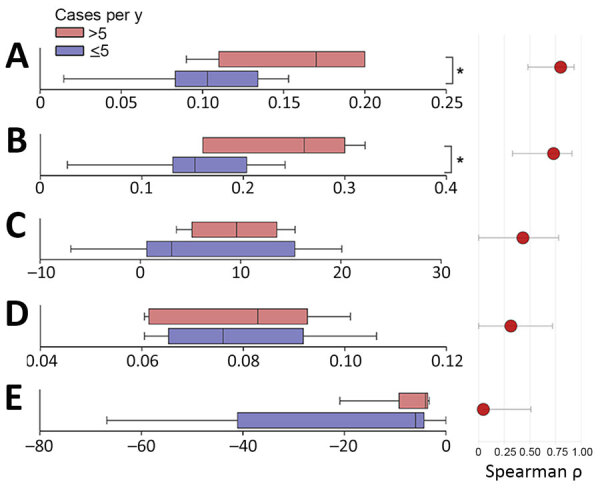
Comparison between ecologic networks parameters in a study of ecologic, geoclimatic, and genomic factors modulating plague epidemics in primary natural focus, Brazil. Graphs compare nonepidemic years (cases ≤5) with epidemic years (cases >5). A) Host robustness; B) vector robustness; C) modularity; D) connectance, which is used to characterize community-wide specialization; E) nestedness based on overlap and decreasing fill. Cutoff value for epidemic status was defined according to the cluster at the principal component analysis. Mann-Whitney test was used to compare groups. Asterisks (*) indicate statistically significant difference (p<0.05). The lower and upper error bars correspond to the first and third quartiles (the 25th and 75th percentiles). Vertical lines within boxes represent medians.

**Figure 10:**
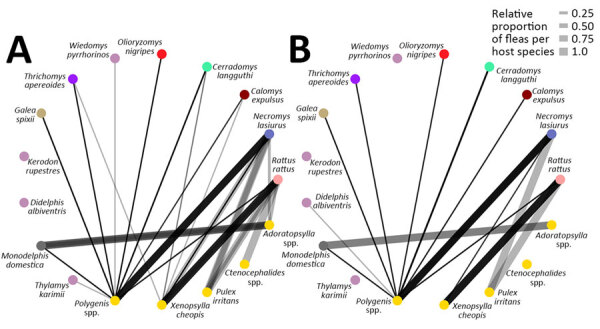
Potential *Yersinia pestis* transmission networks in a study of ecologic, geoclimatic, and genomic factors modulating plague epidemics in primary natural focus, Brazil. A) Epidemic years, >5 human cases; B) nonepidemic years, <5 human cases. Transmission networks were based on biologic interactions between host and vector species. Weight of links represent relative proportion of flea species per small mammal.

The structural properties of the host–vector network exhibited significantly higher robustness in epidemic years than low-activity epidemiologic years (Figure 6), and effects were seen in both small mammal (Mann-Whitney p = 0.014) and flea (Mann-Whitney p = 0.029) groups (Figure 9). Modularity (quantitative modularity) and nestedness (weighted nested overlap and decreasing fill [NODF]) values surpassed those predicted by null models, and appeared similar across epidemiologic years as the connectance, a term used to describe the ratio of observed ecological interactions to the total potential for such interactions. By sorting years according to their epidemiologic status, we observed distinct patterns of biologic interactions between small mammals and flea species (Figure 10, panel A). We also calculated annual values for the ecologic network metrics (Appendix 1 Figure 4, panel A).

Using only epidemiologic variables, 11 of 14 plague years (1977 singularity resulted in an invalid value) were correctly classified by the linear discriminant analysis; 1969 and 1975 were misclassified as not epidemic, and 1976 was misclassified as epidemic. When using 5 different network variable combinations with epidemiologic variables, we found the correct classification of those 14 years was obtained by adding only modularity and nestedness. Those findings showed that the network variables increased the correct discrimination by 21.43% compared with the eco-epidemiologic variables alone. We summarized probabilities of classification for all models by using epidemiologic and network variables, and found that, in terms of predictive probability, 1969 and 1976 were less prone to correct prediction by different models (Appendix 1 Figure 4, panel B).

### Monthly-Based Eco-Epidemiologic Surveillance Parameters as Predictors of Human Plague Risk

**Figure 11:**
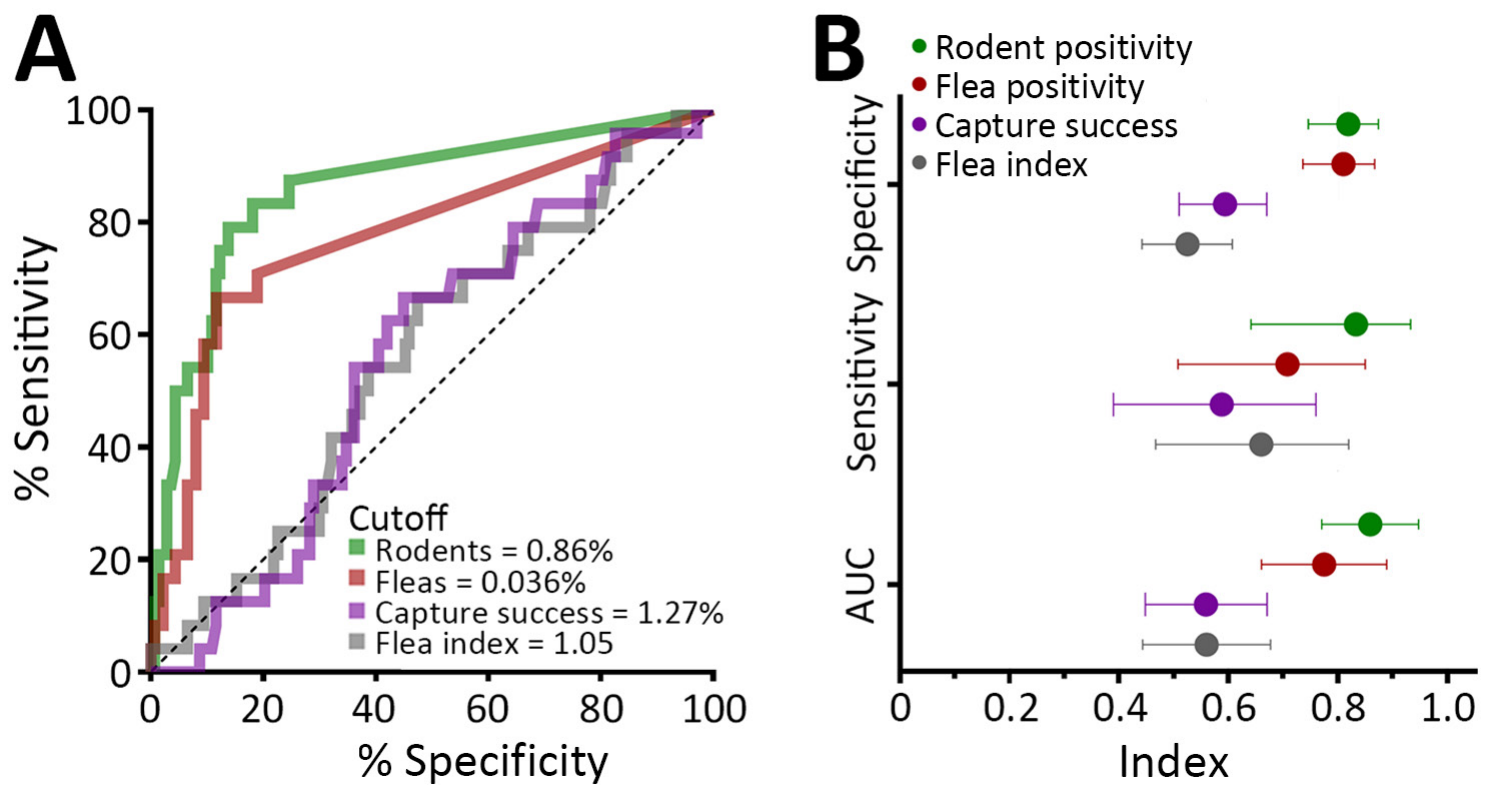
Human risk prediction in a study of ecologic, geoclimatic, and genomic factors modulating plague epidemics in primary natural focus, Brazil. Prediction used ecologic variables at a monthly level. A) Receiver operating characteristic curves and cutoff values of the ecologic variables for the prediction of >2 human cases within the same month. B) Sensitivity, specificity, and AUC for each variable. Error bars indicate 95% CIs. AUC, area under the curve.

To estimate how data from animal surveillance can provide informative alerts of risk for human plague, we next analyzed monthly data. The receiver operating characteristic curve analysis revealed that 0.86% rodent positivity and 0.34% flea positivity are the optimal cutoff values of risk for human cases. We found that rodent and flea *Y. pestis* positivity rates were strong predictors of plague activity in humans in the same month: for fleas, area under the curve was 0.77, sensitivity was 66.7% and specificity was 88.3%; for rodents, area under the curve was 0.86, sensitivity was 83.3%, and specificity was 81.8%. The other ecologic variables, such as capture success and flea index, on the other hand, were poor predictors (Figure 11).

### Genomic Features from *Y. pestis* Isolates

We analyzed 913 *Y. pestis* isolates from human cases, animal reservoirs, and vectors, including 439 isolates from Brazil and 474 from elsewhere. The core genome analysis resulted in a conserved sequence that was equivalent to 46% of the CO92 reference strain (GenBank accession no. GCA_000009065.1). The core SNV contained a total of 1,867 SNV sites that diverged from the CO92 genome.

**Figure 12:**
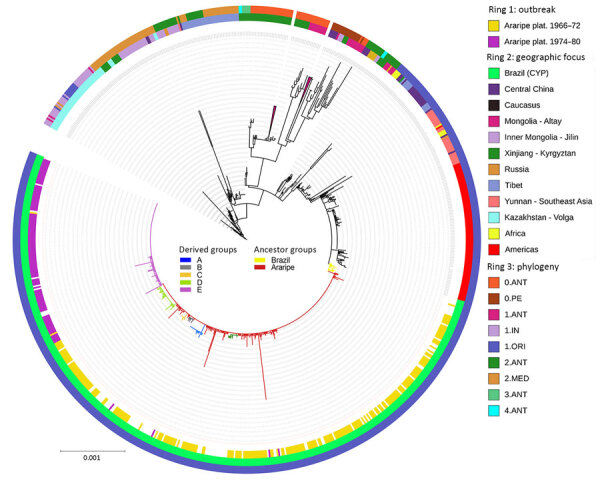
Genomic characterization of *Yersinia pestis* strains in a study of ecologic, geoclimatic, and genomic factors modulating plague epidemics in primary natural focus, Brazil. Phylogenetic tree was based on the 1,867 single-nucleotide variants identified in the core genome from 913 strains isolated in the Araripe Plateau and included in the analysis. The rings contain metadata regarding the epidemiologic features from the Araripe Plateau outbreaks (ring 1), the attributed geographic foci (ring 2), and genetic group provided in the NextStrain dataset (https://nextstrain.org) (ring 3). Brazil branches are colored according to their genetic subgroups. Scale bar indicates nucleotide substitutions per site.

The core SNV approach identified discernible patterns of geographic distribution of *Y. pestis* lineages at the global level. Strains from Brazil were a relatively homogeneous genetic group, assigned within the 1.ORI phylogenetic population (Figure 12). Isolates from Brazil constituted a monophyletic clade that derived directly from strains from South America and North America, which in turn branched from strains from Yunnan and Southeast Asia. That genetic connection sheds light on the historical dissemination of *Y. pestis* in Brazil. Among *Y. pestis* genomes from the NextStrain dataset (https://nextstrain.org), strains from the same geographic plague foci grouped together within the phylogenetic tree (Figure 12).

The plague spikes in Brazil during 1966–1972 were constituted by a basal occurrence of the main, undifferentiated, genetic group that we termed Araripe ancestor (Figure 12), which derived some transitory groups that were unsuccessful in replacing the basal lineage over time. One of those genetic groups, group D, was isolated in August 1969 and quickly expanded, representing 76% (28/37) of *Y. pestis* isolates during October 1969–March 1970, when the cases in the region ceased. In the second semester of 1970, when plague started to disseminate again, we did not identify any group D strains, and we assigned the new isolates to the Araripe ancestor group.

**Figure 13:**
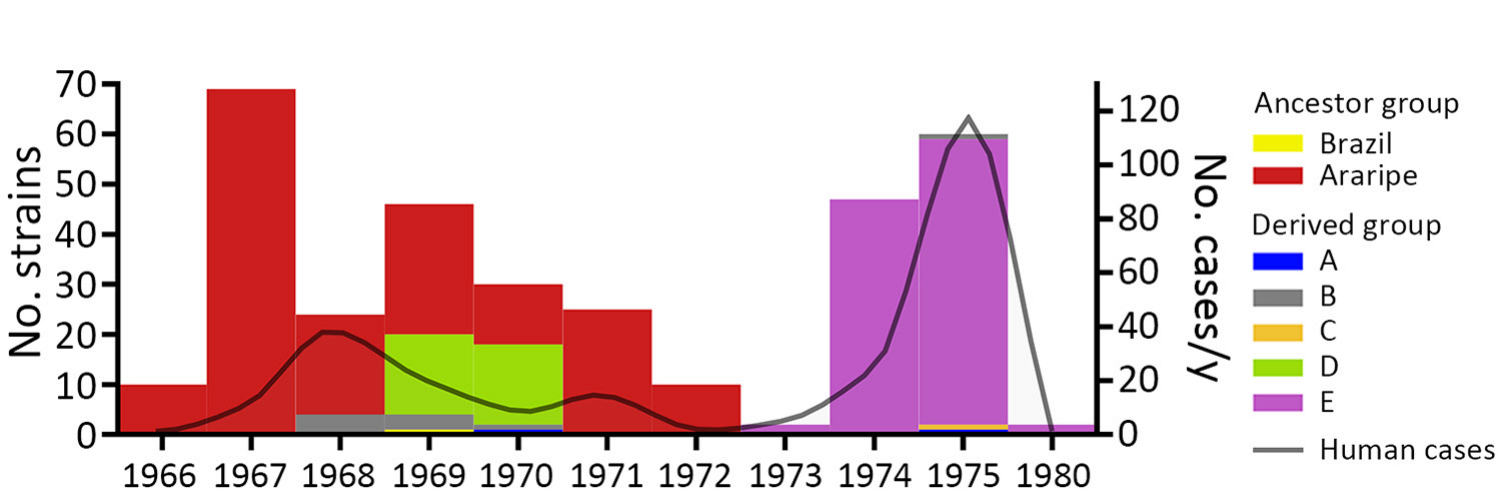
Temporal distribution of *Yersinia pestis* strains in a study of ecologic, geoclimatic, and genomic factors modulating plague epidemics in primary natural focus, Brazil. The graph shows genomic characterization of *Y. pestis* strains from this study compared with the number of human plague cases per year. Scales for the y-axes differ substantially to underscore patterns, but do not permit direct comparisons.

**Figure 14:**
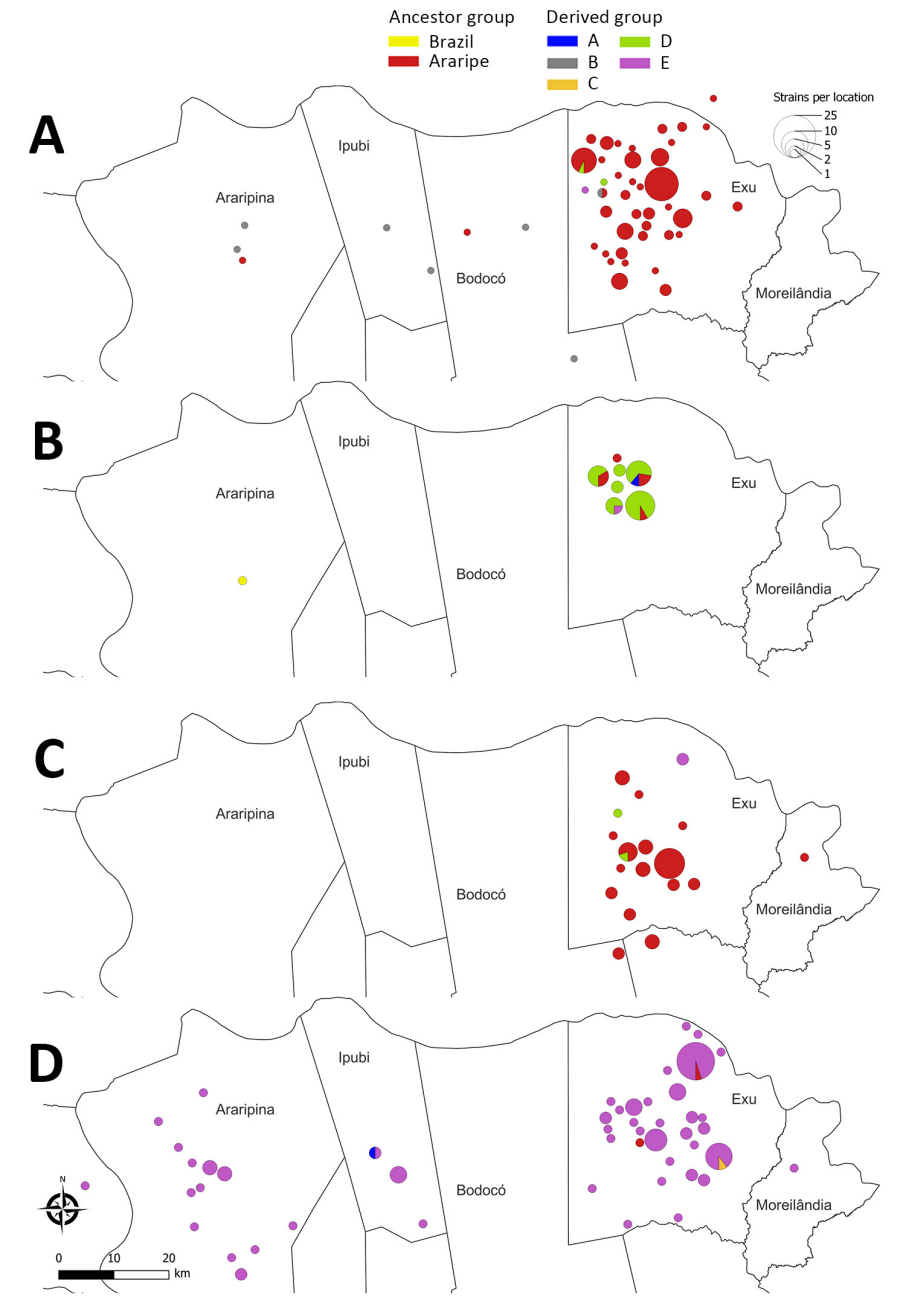
Spatial distribution of *Yersinia pestis* strains in a study of ecologic, geoclimatic, and genomic factors modulating plague epidemics in primary natural focus, Brazil. The sequence strains from the Araripe Plateau are shown during various timeframes: A) August 1966–October 1969; B) November 1969–March 1970; C) April 1970–May 1973; and D) June 1973–December 1980. Detailed spatiotemporal dynamics of *Y. pestis* lineages in the Araripe Plateau are available at https://microreact.org/project/fNz1zKcNyCTmKQrtke33Gm-yersinia-pestis-strains-in-the-araripe-plateau-brazil.

The *Y. pestis* strains isolated during the 1974–1975 outbreak formed a monophyletic and discernible clade, group E (Figure 12). Of note, the only 2 isolates during the quiescent year of 1973 were group E, which was uncommon in previous years. After 1973, however, that clade promptly replaced the previous strains circulating in the region, which overlapped with an unprecedented upsurge of plague cases (Figure 13). In addition, the lineage replacement caused by group E strains was observed temporally and spatially (Figure 14). We noted the apomorphic mutations that defined each of those derived groups, along with their predicted effects on protein function (Appendix 2 Table 1).

## Discussion

We aimed to dissect plague epidemics that occurred in the Araripe Plateau focus of Brazil. By combining epidemiologic, ecologic, climatic, and genomic information, we were able to learn relevant aspects of the intricate spatiotemporal dynamics of plague in the region. Analysis from the temporal series showed that eco-epidemiologic and climatic factors had a stark and linear influence on the risk for human plague. Monthly rodent and flea positivity rates strongly indicated the risk for human cases and cutoff values were <1% positivity, meaning any basal detection of *Y. pestis* bacteria in wildlife signaled a risk for human plague cases. Although these indicators have high sensitivity and specificity, detecting early-stage risk requires a large number of sampled animals. Those findings support previous studies suggesting that serosurveys in sentinel species, such as dogs, are more efficient and require fewer tests (*12*).

Human plague in the Araripe Plateau region typically started at the end of the rainy season and peaked in the driest months, August–September. Our results showed that rainfall affected plague dynamics in this semiarid region by modulating the wild rodent and flea abundance. Sylvatic rodent and flea populations expand after the rainy season until the peak of the dry season, and once food and water sources become scarce, rodents carrying vector fleas are attracted to human domestic perimeters by the grain crops grown there. Those events overlap the quick dissemination of plague in the rodent communities, leading to rodent dieoffs and the search for new hosts by the fleas.

Precipitation patterns have been implicated in plague risk in distinct ecosystems (*4*,*13*–*15*). Our findings suggest that rainfall could set the ground conditions for plague dissemination in the following year. Similarly, studies from other plague systems also reported a lagged effect of precipitation on plague risk (*15*–*17*). Of note, the plague epidemic described here overlapped with a period of cool and negative Pacific decadal oscillation teleconnection that lasted until 1976 (*18**–**20*), which is supported by previous studies performed in other ecosystems (*17*).

Geospatial analysis revealed that locations at lower altitudes and closer to the concave sectors from the plateau were at higher risk for plague occurrence. We hypothesized that those locations provided specific conditions that led to the dissemination of plague in wildlife and, eventually, in humans. That hypothesis was further supported by the marked spatial overlap between plague hotspots with increased rainfall and vegetation coverage areas. Of note, although the top of the plateau has savanna-like vegetation, the concave slopes are described to encompass unique characteristics, including forest vegetation and increased water availability due to orographic rain (*7*,*21*). Those findings support the trophic cascade hypothesis and underscore the influence of climate factors, such as water availability, vegetation coverage, and host–vector abundance, on *Y. pestis* dissemination. Similar patterns have been observed in other systems, indicating a broader ecologic context where climate is implicated in the spread of plague (*14*,*17*,*22*). Identifying well-defined hotspots with conditions optimal for plague outbreaks is crucial for maximizing an assertive epidemiologic surveillance of this neglected yet reemerging disease, especially in resource-constrained settings.

The scarcity of satellite-derived data throughout the period studied restricted our access to variables essential for modeling the suitability of plague resurgence. Moreover, the abrupt disappearance of plague after a massive rodent dieoff in 1975 potentially could be attributed to the depletion of sensitive hosts or vectors and the expansion of resistant ones (*22*,*23*). Consequently, further research encompassing later timeframes and integrating satellite-derived climatic, topographic, and land-use data, alongside assessments of host–vector suitability for *Y. pestis* infection, is imperative for accurately modeling potential scenarios of plague resurgence.

Beyond its effects on human health, sylvatic plague raises major conservation concerns. Our findings show that plague activity correlated with substantial declines in abundance of *N. lasiurus* mice. The drastic effect on wild rodent abundance has been reported to cascade through the ecosystem, affecting prey–predator balance and consequently, biodiversity and ecosystem stability (*24*). Furthermore, plague can disturb ecologic systems by directly infecting a wide range of mammal species (*25*,*26*). In this study, the presence of generalist species with extensive interactions contributed substantially to the network’s robustness during epidemic years; however, a sharp population decline in those species impacts most of their interaction partners, rendering generalist species surveillance crucial (*27*,*28*). We found the potential for plague maintenance via multiple species interactions, akin to patch dynamics (*29*), is especially evident during epidemic years. Such periods exhibited greater stability in maintaining transmission pathways despite local species extinctions, which was evidenced by higher network robustness in epidemic years.

The amalgamation of phylogenetic and spatiotemporal characteristics in *Y. pestis* isolates from the Araripe focus highlighted the incidence of a foundational ancestral strain. That strain exhibited the capability to evolve into distinct genetic groups, each linked to temporally and spatially constrained outbreaks. Of note, the large outbreak of 1974–1975 was observed in immediate succession to the displacement of previously prevalent *Y. pestis* strains by a singular clade. The expansion of specific *Y. pestis* clades temporarily overlapped with 2 atypical years, 1969 and 1976, in the eco-epidemiologic linear model. Phylogroup D appeared in 1969, and 1976 marked the aftermath of the largest plague outbreak period in the region; strains from that outbreak were genetically characterized by displacement of prior lineages by phylogroup E.

The results from this study rely on records generated during epidemiologic surveillance of human and animal plague. The first limitation of this study is the lack of case-level data in the state of Ceará in the northern range of the Araripe Plateau, which limited most data analysis to the southern part in Pernambuco state. The second limitation is that, although plague surveillance in rodents and fleas was uninterrupted during the study period, outbreaks in specific sites might have biased the rodent capture effort toward specific locations. Finally, because satellite images were available only after the study period, a 4-year delay occurs between surveillance and NDVI representation.

In conclusion, this study identified well-delimited pockets of plague activity spanning areas just a few kilometers wide in the Araripe Plateau region of Brazil. Through the lens of One Health, we determined plague hotspots by using a specific combination of rainfall, vegetation, and landscape features. We also measured the impact of plague activity on wild rodent populations and spillover events in humans. Our research provides a holistic understanding of the ecologic implications of plague in an ecosystem of Brazil. Our findings from Araripe Plateau data could help refine plague risk areas and improve surveillance in other plague foci within the Caatinga biome.

Appendix 1 Additional information on methods used for a study of ecologic, geoclimatic, and genomic factors modulating plague epidemics in primary natural focus, Brazil.

Appendix 2 Genomic sequences from a study of ecologic, geoclimatic, and genomic factors modulating plague epidemics in primary natural focus, Brazil.

Appendix 3 Metadata on *Yersinia pestis* sequences from a study of ecologic, geoclimatic, and genomic factors modulating plague epidemics in primary natural focus, Brazil.
