# Supplementary figures and images for: Ecologic, Geoclimatic, and Genomic Factors Modulating Plague Epidemics in Primary Natural Focus, Brazil
Source: Emerg Infect Dis. 2024 Sep;30(9):1850–64. doi: 10.3201/eid3009.240468 (PMC11346973; doi:10.3201/eid3009.240468)

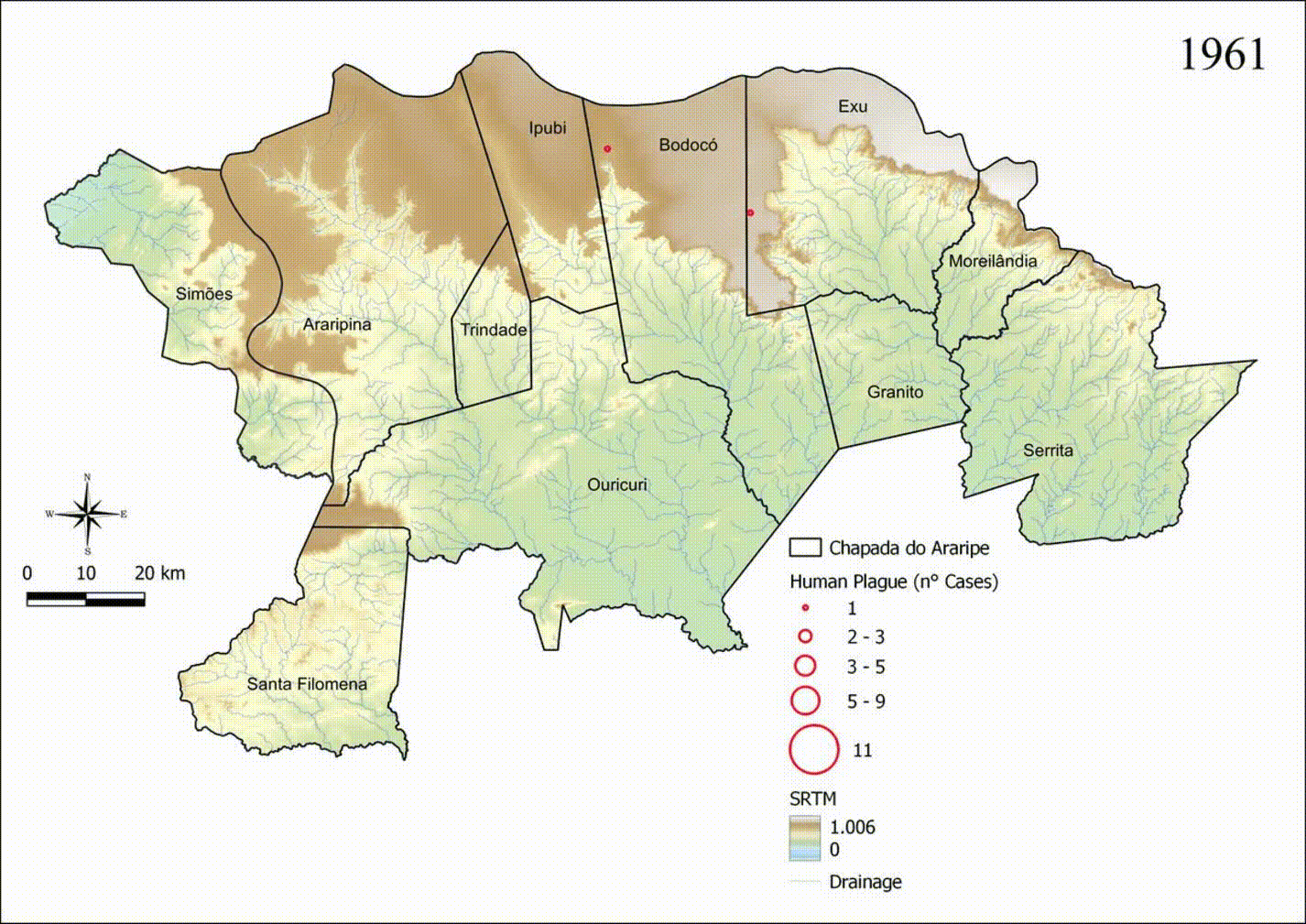

Supplement: Supplementary file 1 [file 24-0468-V.gif]
