# Supplementary material for: Ecologic, Geoclimatic, and Genomic Factors Modulating Plague Epidemics in Primary Natural Focus, Brazil
Source: Emerg Infect Dis. 2024 Sep;30(9):1850–64. doi: 10.3201/eid3009.240468 (PMC11346973; doi:10.3201/eid3009.240468)
Supplement: Appendix 1 — Additional information on methods used for a study of ecologic, geoclimatic, and genomic factors modulating plague epidemics in primary natural focus, Brazil. [file 24-0468-Techapp-s1.pdf]

Article DOI: https://doi.org/10.3201/eid3009.240468

EID cannot ensure accessibility for supplementary materials supplied by authors. Readers who have difficulty accessing supplementary content should contact the authors for assistance.

Ecologic, Geoclimatic, and Genomic Factors Modulating Plague Epidemics in Primary Natural Focus, Brazil

Appendix 1

Additional Methods

Data Collection

We combined multiple data sources, including reservoirs and vector laboratorial surveillance, notification of human cases, the *Yersinia pestis* Fiocruz/CYP biologic collection (WDCM accession number: 1040), CONCEPAS database, and climatic/environment variables from public databases (Appendix 1 Figure 1). The details from each dataset are provided below. Information regarding human plague cases in the Araripe plateau region from 1961 to 1980 was acquired through the examination of Human Plague Occurrence notification forms, which are maintained at the National Plague Control Program's documentary collection. Notably, municipalities in Pernambuco and Piauí provided comprehensive details, including individual-level data such as the date of case notification/disease onset and geographic coordinates. In contrast, municipalities in the state of Ceará were aggregated annually for each municipality.

Data from the reservoir/vector epidemiologic surveillance (1966–1980) included rodent capture success (a proxy of rodent abundance), rodent species relative abundance and their ectoparasites, rodents and flea infection positivity index, average number of fleas per individual (flea index). These data were obtained by consulting the original laboratory registry books. Information regarding the *Y. pestis* strains isolated in the region was extracted from the CONCEPAS database (http://cyp.fiocruz.br/index?services).

The precipitation data were extracted from the Water and Climate agency from Pernambuco state (Agência Pernambucana de Águas e Climas - APAC,

https://www.apac.pe.gov.br). The yearly averages were calculated as the average of monthly averages from the given year. Monthly average was determined by the average values of the measured water volume from all the meteorological stations available in the region. For the seasonal analysis, we calculated the average values for each month considering the study timeframe (1961–1980). The list of all meteorological stations used in the study is provided in Appendix 2 Table 3. Importantly, the pluviosity-related analyses are limited to the portion of the Araripe plateau located in the Pernambuco state because there was poor data availability for Ceará and Pauí states during the study timeframe.

Laboratory Testing for Plague

The captured rodents were maintained in quarantine cages and were observed on a daily basis. The individuals that died during quarantine were immediately tested for plague and the survivors were tested 4 weeks after capturing. The laboratorial diagnosis in rodents during the PPP program was performed through *Y. pestis* detection by direct microscopy observation of spleen imprints and blood smears, followed by conventional bacteriology as previously described (*1*). Suspected colonies were confirmed with the bacteriophage test (*2*). The fleas were also tested in bacterial culturing. The diagnosis in humans was performed through the combination of clinical and epidemiologic assessment and laboratory testing (bubo aspirates and blood cultures).

Geoprocessing

Study area: The geographic scope encompasses the plateau, characterized by an expansive summit plateau situated at an elevation of 950 m above sea level. The plateau is surrounded by scalloped escarpments undergoing noticeable erosive retreat, featuring a topographical disparity of 250 m in the extreme west (Araripina county) and 500 m in the east (Exu and Moreilândia counties).

For the production of the georeferenced maps, coordinates were collected with on-site visits. Locations of interest were georeferenced using the global positioning system (GPS) technology, using a GPS receiver model eTrex Vista Cx, Garmin (Kansas City, USA), UTM projection, Datum SIRGAS 2000, with a minimum accuracy of 10 m. The Cartographic Base (limit of municipalities, districts, census tracts, subnormal agglomerations), data on the estimated population and hydrography information were obtained from the Brazilian Institute of Geography and Statistics (IBGE - http://www.ibge.gov.br).

Kernel Density Estimation (KDE) was constructed, using the shapefile from the locations where human cases of plague occurred, the radius was calculated from the matrix of distance of the points and the mean and standard deviation (SD) values, applied to the following equation: $R = \bar{x} \pm \bar{x}\sigma$. The calculated value was weighted by the number of cases per location. Incidence map was calculated as follows: the total number of plague cases in the period from 1961 to 1980 per municipality divided by the population in the middle of the period (1970), adjusted for 100,000 inhabitants/year. The space-time scan statistics (https://www.satscan.org) used the discrete Poisson model. To increase the granularity of the analysis, the municipalities were subdivided into smaller polygons, called census tracts (n = 268). The reference population size was that of the 2000 census (first year in which the population was recorded at a census tracts level). For the construction of pluviosity maps, the rainfall data described above was interpolated using the Inverse Weighted Distance - IDW method, in the linear interpolation and quartile mode.

The Normalized Difference Vegetation Index (NDVI) map was constructed using the mosaic of satellite images that represents the extension of the territory of Araripe Plateau. The formula NDVI = (NIR - red) / (NIR + red) was used, where NIR is near infrared and red: visible red light spectrum. LandSat4 images were downloaded from the GloVis-USGS database (https://earthexplorer.usgs.gov). The hydrography was obtained from the Mineral Resources Research Company (CPRM) (https://www.cprm.gov.br/en/Hydrology-83) and the Digital Elevation Model (DEM) data was obtained from the Shuttle Radar Topography Mission (SRTM) refined for the Brazilian territory from the original resolution of 3 arc seconds to 1 arc second using a geostatistical approach (http://www.dsr.inpe.br/topodata).

Ecologic Networks

We constructed *Y. pestis* potential transmission networks based on interactions between small mammal species (reservoirs) and the fleas (vectors) captured during plague surveillance activities between 1966–1980 in the Araripe plateau. A network was structured for each year, with nodes representing small mammals and fleas species, and links denoted the relative proportion of fleas species per small mammals species, resulting in 15 networks.

We analyzed the fundamental properties of each host-vector plague network using the bipartite R package (*3*). The structural properties of each network were analyzed temporally

through the examination of connectance, which realized the proportion of possible interactions between fleas and small mammals species (*4*). We tested for a nested arrangement of the networks using the quantitative method weighted NODF (*5*). Network robustness (*6*) were estimated by quantifying the weighted loss of interactions between fleas after randomly removing species of small mammals, and vice versa. We searched for groups of species that interact more inside the group than outside using the Q modularity metrics (*7*). Due to variations in network size, modularity and nestedness were standardized to z-scores. This involved comparing observed values minus the mean value estimated by null models, divided by the standard deviation of null models, using the *vaznull* null model method (*8*).

We anticipated higher connectance and link density during epidemic years, potentially amplifying transmission probabilities. Furthermore, the presence of generalist and abundant species was expected to boost nestedness values during epidemics, thereby promoting plague transmission within the network. We hypothesized that more modular networks, by limiting connections into distinct groups, would reduce transmission flow during low activity years. Additionally, we expected higher network robustness in epidemic years, as they maintain more connections even after species loss. When a species was removed from epidemic networks, it was expected that other generalist species would compensate for the interactions without losing connections within the subset.

The network's structural metrics were correlated with the epidemiologic status of the year (Epidemic >5 human plague cases; non-epidemic ≤5 human plague cases) to test for the impact of network properties on the outbreaks.

Statistical Analysis of Eco-Epidemiologic Data

Comparison between the epidemiologic status of the year with continuous variables was performed by linear regression. The R-squared for the rodents' capture success were calculated using an exponential (one-phase decay) nonlinear regression. Principal component analysis (PCA) was calculated selecting PCs based on eigenvalues method. Regressions, PCA and Receiver Operating Characteristic (ROC) curves were calculated using GraphPad Prism v10.1 (Dotmatics, Boston, USA). The Sankey diagram was designed using the Sankey Diagram Generator (http://sankey-diagram-generator.acquireprocure.com). Statistic tests were run using a 95% confidence interval and considered significant when $p<0.05$.

Linear Discriminant Analysis

To test the contribution of variables to the prediction of plague epidemics by year, the years were assigned to groups according to the results from the PCA analysis: epidemic years (when human cases >5) and non-epidemic years (when human cases ≤5). We carried a linear discriminant analysis (LDA) with cross validation with different combinations of the epidemiologic variables (Rodent capture success, Flea positivity, Rodent positivity, Flea index) and network variables (modularity, vector robustness, host robustness). The discriminant analyses were carried only with the epidemiologic variables and with all combinations of network variables. A contingency table was obtained based on the different combination of variables which years were classified as epidemic and not epidemic as well as the probability of classification of each year.

Whole-Genome Sequencing

The *Y. pestis* cultures isolated in the Araripe Plateau are maintained in the cultures collection (Fiocruz/CYP) of the SRP from the IAM. In previous studies, 407 cultures were recovered and their genomes sequenced and analyzed (*9*,*10*). Here we revived and sequenced 33 additional strains that were originally contaminated, using the CYP broth medium (*11*).

DNA extraction was carried out using the DNeasy Blood & Tissue Kit (Qiagen) in accordance with the manufacturer's recommendations. Subsequently, the extracted DNA was quantified using the Qubit® 4 Fluorometer and the Qubit dsDNA High Sensitivity Kit (Thermo Fisher Scientific Inc.). Genomic library preparation was conducted using the Nextera XT Library Preparation Protocol (Illumina Inc.). Subsequently, library quantification and normalization were conducted using the ProNex® NGS Library Quant Kit (Promega) through Quantitative Real-Time PCR (qPCR) for standardized sample input using the 7500 Fast Real-Time PCR System (Applied Biosystems). After quantification, the samples were diluted to 19 pmol in ultrapure water and transferred to a single microtube containing Hybridization Buffer HT1 (Illumina Inc.). Subsequently, they were denatured at 96°C for 2 minutes in a thermocycler and a total of 600 µL of the denatured samples was loaded into a MiSeq Reagent Kits v3 (Illumina Inc.).

Genome Assembly, Annotation, and Core Genome

The quality of the sequencing data of the 439 Brazilian strains of *Y. pestis* was evaluated using FastQC 0.11.8 and the results were grouped by MultiQC v1.10 as previously described

(*12*,*13*). The sequencing metrics and metadata for the 33 newly sequenced Brazilian strains are available in Appendix 2 Tables 4, 5. Sequencing data was filtered using Trimmomatic v. 0.38 (*14*) and the assembly was performed using the VelvetOptimiser. To perform gene predictions and functional annotations of the newly assembled genomes, the Prokka pipeline (*15*) was used as described by Pitta *et al.* (*9*). GenomeTools 1.5.8 (*16*) was used to evaluate the annotations performed.

SNV Calling and SNV Core Genome

We conducted SNV calling with the Snippy software (*17*) and the CO92 strain was used as a reference genome. The results obtained were subsequently employed to generate a core SNV alignment, which served as the basis for the subsequent steps involved in constructing a phylogenetic analysis. Given the substantial volume of genomes, the 'snippy-multi' script (an integral component of the Snippy package) was employed for the automated analysis of all genomes in comparison to the reference genome.

Phylogenetic Analyses

The alignments were used as input for IQ-TREE2 (*18*) to perform phylogenetic analyses based on the core SNV profile with a bootstrap value of 1,000 and the ultrabootstrap approximation technique to enhance computational efficiency and reduce analysis time. Additionally, the ModelFinder tool (*19*), integrated into IQ-TREE2, was employed to automatically assess the best substitution model for the phylogenetic analysis. The results were visualized with the iTOL (Interactive Tree of Life) web platform (*20*).

NextStrain Dataset

We retrieved from the NextStrain dataset 538 *Y. pestis* genomes (filters: samples dated later than 1900 and not from Brazil). The metadata from the web service was subsequently downloaded, and based on the bioproject associated with each sample, a list for the download of these genomes was generated through the NCBI web service. Out of the 538 genomes, 64 are no longer available on NCBI; therefore, a total of 474 *Y. pestis* genomes were successfully downloaded (Appendix 3). A variable regarding the geographic focus was created for the phylogenetic analysis.

Characterization of the Phylogenetic Subgroups SNVs

Based on the variant call file (VCF) generated by Snippy during the core SNP analysis and the phylogeny constructed from this analysis, we identified which the apomorphic SNVs for each of the phylogenetic subgroups deriving from the "Araripe ancestor" group. Subsequently, SnpEff was used to predict the functional impacts of these variants in the highlighted samples, focusing on changes such as amino acid substitutions. The mutation list is available in Appendix 2 Table 1.

References

1. Bahmanyar M, Cavanaugh DC. Plague manual. Geneva: World Health Organization; 1976.

2. Karimi Y. Rapid laboratory diagnosis of plague [in French]. Bull Soc Pathol Exot. 1978;71:45–8.

3. Dormann CF, Fründ J, Blüthgen N, Gruber B. Indices, graphs and null models: analyzing bipartite ecological networks. The Open Ecology Journal. 2009;2:12917–22. https://doi.org/10.2174/1874213000902010007

4. Dunne JA, Williams RJ, Martinez ND. Food-web structure and network theory: The role of connectance and size. Proc Natl Acad Sci U S A. 2002;99:12917–22. PubMed https://doi.org/10.1073/pnas.192407699

5. Almeida-Neto M, Guimarães P, Guimarães PR Jr, Loyola RD, Ulrich W. A consistent metric for nestedness analysis in ecological systems: reconciling concept and measurement. Oikos. 2008;117:1227–39. https://doi.org/10.1111/j.0030-1299.2008.16644.x

6. Memmott J, Waser NM, Price MV. Tolerance of pollination networks to species extinctions. Proc Biol Sci. 2004;271:2605–11. PubMed https://doi.org/10.1098/rspb.2004.2909

7. Beckett SJ. Improved community detection in weighted bipartite networks. R Soc Open Sci. 2016;3:140536. PubMed https://doi.org/10.1098/rsos.140536

8. Vázquez D, Melian CJ, Williams NM, Blüthgen N, Krasnov BR, Poulin R. Species abundance and asymmetric interaction strength in ecological networks. Oikos. 2007;116:1120–7. https://doi.org/10.1111/j.0030-1299.2007.15828.x

9. Pitta JLLP, Bezerra MF, Fernandes DLRDS, Block T, Novaes AS, Almeida AMP, et al. Genomic analysis of *Yersinia pestis* strains from Brazil: search for virulence factors and association with epidemiological data. Pathogens. 2023;12:991. PubMed https://doi.org/10.3390/pathogens12080991

10. Vogler AJ, Sahl JW, Leal NC, et al. A single introduction of *Yersinia pestis* to Brazil during the 3rd plague pandemic. PLoS One. 2019;14:e0209478. PubMed https://doi.org/10.1371/journal.pone.0209478

11. Rocha IV, Andrade CAN, Sobreira M, Leal NC, Almeida AMP, Bezerra MF. CYP broth: a tool for *Yersinia pestis* isolation in ancient culture collections and field samples. Appl Microbiol Biotechnol. 2023;107:2653–60. PubMed https://doi.org/10.1007/s00253-023-12452-0

12. Andrews S. FastQC: a quality control tool for high throughput sequence data [cited 2020 Apr 21]. http://www.bioinformatics.babraham.ac.uk/projects/fastqc

13. Ewels P, Magnusson M, Lundin S, Käller M. MultiQC: summarize analysis results for multiple tools and samples in a single report. Bioinformatics. 2016;32:3047–8. PubMed https://doi.org/10.1093/bioinformatics/btw354

14. Bolger AM, Lohse M, Usadel B. Trimmomatic: a flexible trimmer for Illumina sequence data. Bioinformatics. 2014;30:2114–20. PubMed https://doi.org/10.1093/bioinformatics/btu170

15. Seemann T. Prokka: rapid prokaryotic genome annotation. Bioinformatics. 2014;30:2068–9. PubMed https://doi.org/10.1093/bioinformatics/btu153

16. Gremme G, Steinbiss S, Kurtz S. GenomeTools: a comprehensive software library for efficient processing of structured genome annotations. IEEE/ACM Trans Comput Biol Bioinformatics. 2013;10:645–56. PubMed https://doi.org/10.1109/TCBB.2013.68

17. Seemann T. snippy: fast bacterial variant calling from NGS reads [cited 2023 Sep 5]. https://github.com/tseemann/snippy

18. Minh BQ, Schmidt HA, Chernomor O, Schrempf D, Woodhams MD, von Haeseler A, et al. IQ-TREE 2: new models and efficient methods for phylogenetic inference in the genomic era. Mol Biol Evol. 2020;37:1530–4. PubMed https://doi.org/10.1093/molbev/msaa015

19. Kalyaanamoorthy S, Minh BQ, Wong TKF, von Haeseler A, Jermiin LS. ModelFinder: fast model selection for accurate phylogenetic estimates. Nat Methods. 2017;14:587–9. PubMed https://doi.org/10.1038/nmeth.4285

20. Letunic I, Bork P. Interactive Tree Of Life (iTOL) v4: recent updates and new developments. Nucleic Acids Res. 2019;47:W256–9. PubMed https://doi.org/10.1093/nar/gkz239

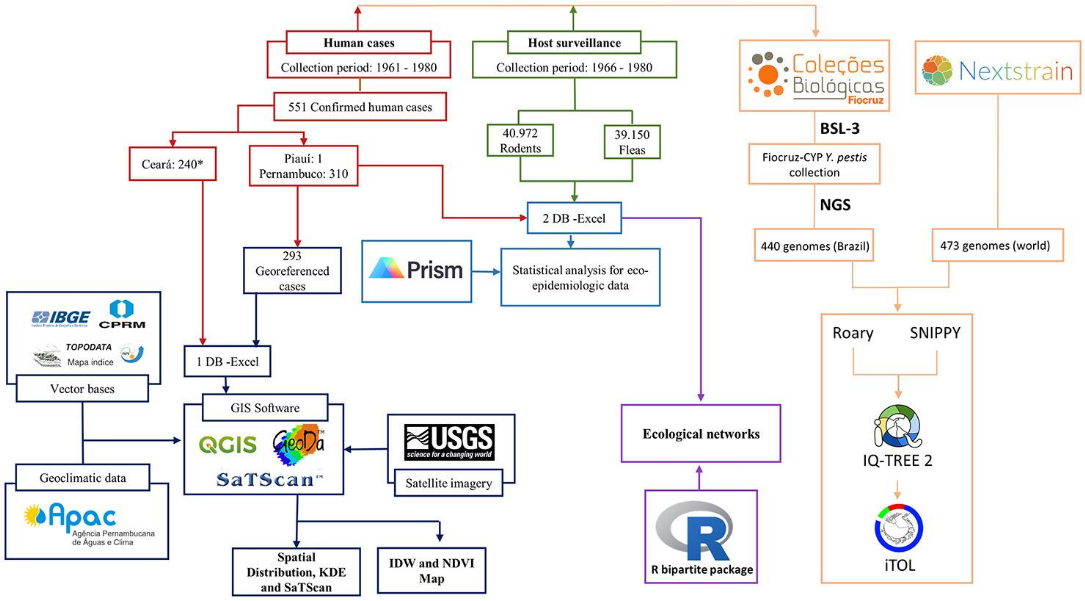

Appendix 1 Figure 1. Summary of the study workflow. The study uses multiple data sources including epidemiologic, ecologic, geospatial, climatic and genomic data. *Data from human cases from Ceará is limited to the total amount per municipality per year.

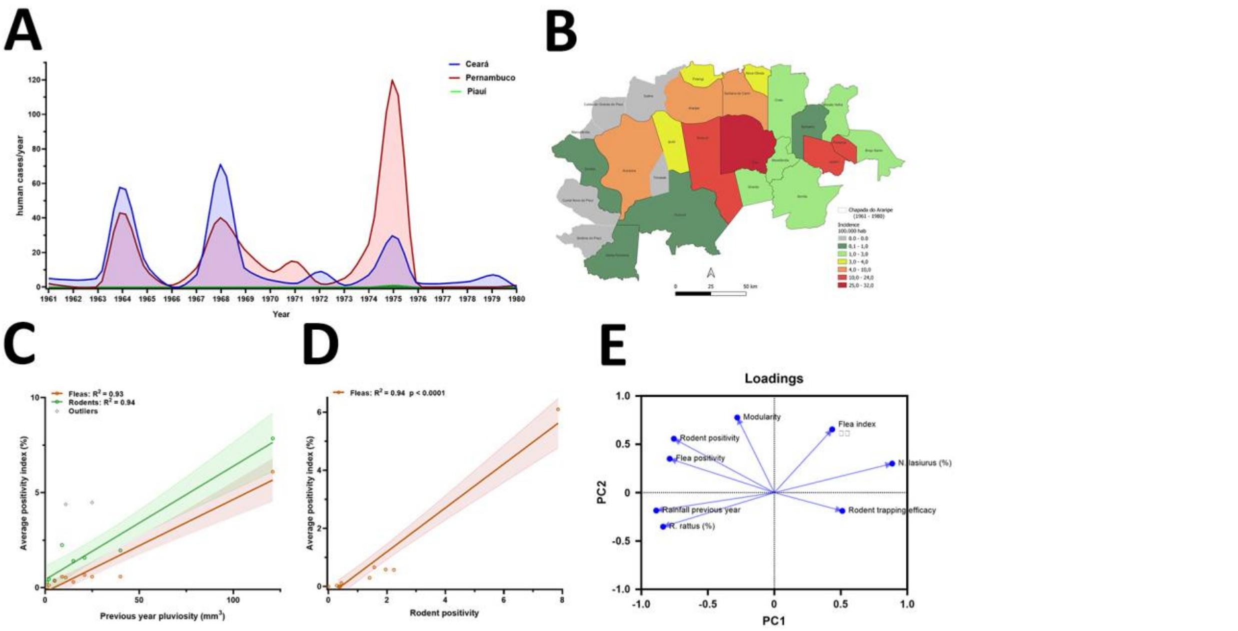

Appendix 1 Figure 2. Human cases of plague in the Araripe Plateau and support information for Figure 2. A) Plague cases according to the state (Pernambuco, Ceará, and Piauí). B) Colormap showing the average plague incidence per municipality (1961–1980). C) Correlation between flea and rodent positivity rate with previous year pluviosity. D) Correlation between flea and rodent *Yersinia pestis* positivity rates. E) Loading plot of the PC1 and PC2, complementary to the PCA plot in Figure 5.

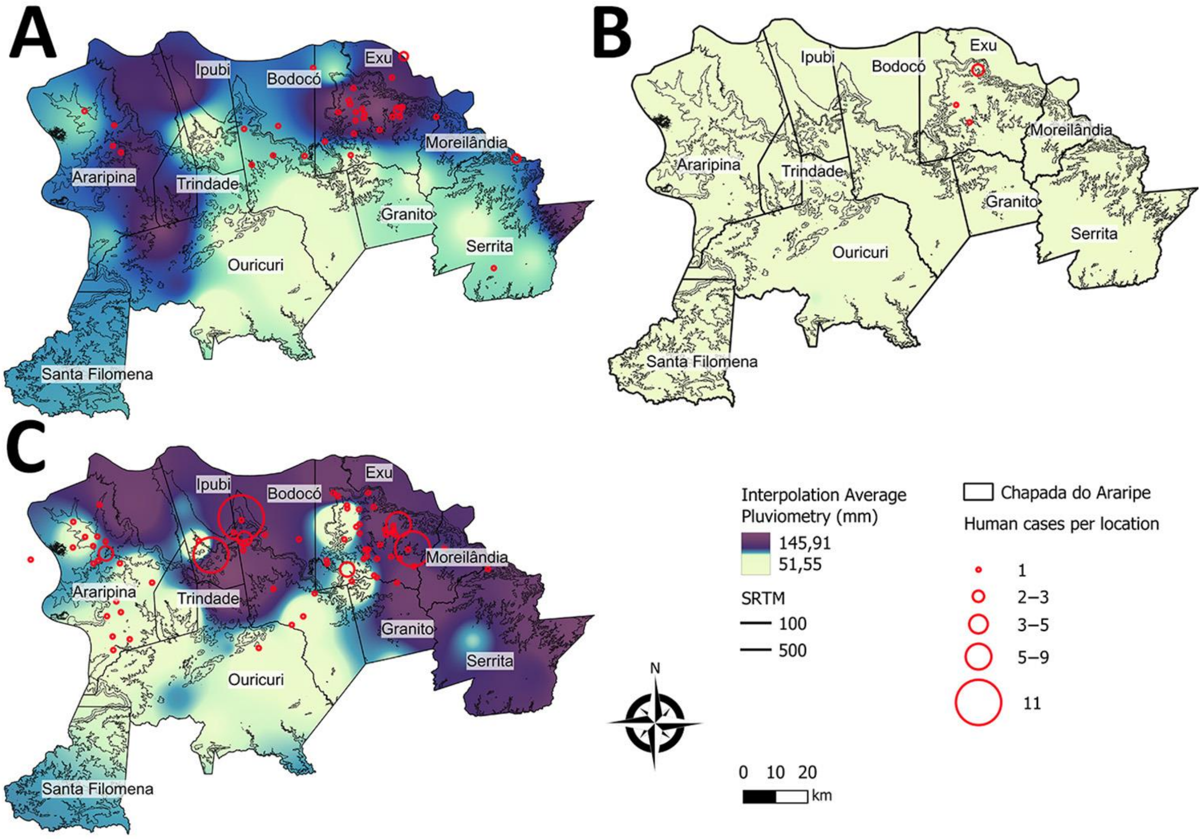

Appendix 1 Figure 3. Pluviometry (IDW Interpolation) and human cases of plague in Araripe Plateau focus in Pernambuco. A) Spatial distribution of the 1967 average rainfall and 1968 plague cases. B) 1972 average rainfall and 1973 plague cases and C) 1974 average rainfall and 1975 plague cases.

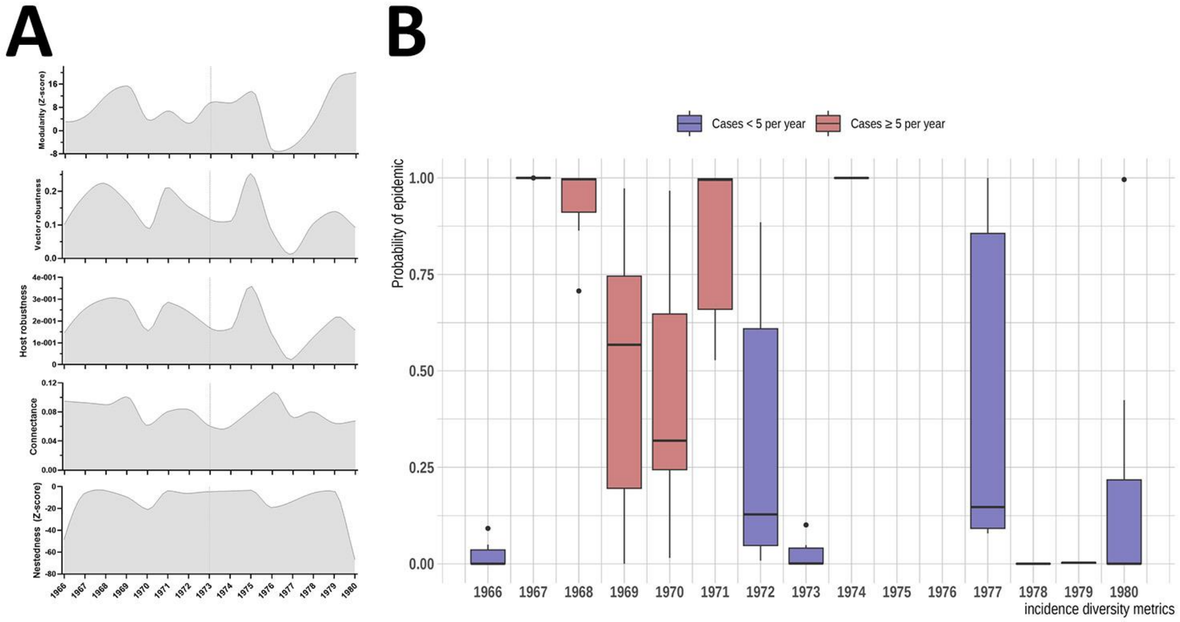

Appendix 1 Figure 4. Annual variation of ecologic networks metrics and LDA. A) Each plot describes one aspect of the ecologic networks in the Araripe Plateau during the study period. The values were calculated based on the relative proportion of interactions of fleas in small mammal species. B) Linear discriminant analysis with cross validation with different combinations of the epidemiologic variables. The bars show the probabilities of classification for all models. The lower and upper hinges correspond to the first and third quartiles (the 25th and 75th percentiles). The whiskers extend from the hinge to the largest/smallest values no further than 1.5 interquartile ranges and outliers are plotted individually.
